# Germline DNA damage response gene mutations as predictive biomarkers of immune checkpoint inhibitor efficacy

**DOI:** 10.3389/fimmu.2024.1322187

**Published:** 2024-01-29

**Authors:** Michael J. Dennis, Sophia Bylsma, Lisa Madlensky, Meghana S. Pagadala, Hannah Carter, Sandip P. Patel

**Affiliations:** ^1^ Division of Medical Oncology, Moores Cancer Center, University of California, San Diego, San Diego, CA, United States; ^2^ Division of Head and Neck Oncology, Dana-Farber Cancer Institute, Boston, MA, United States; ^3^ School of Medicine, University of California, San Diego, San Diego, CA, United States; ^4^ Division of Genomics and Precision Medicine, University of California, San Diego, San Diego, CA, United States; ^5^ Department of Medicine, University of California, San Diego, San Diego, CA, United States

**Keywords:** immune checkpoint inhibitor, germline, biomarkers, DNA damage response, tumor-agnostic, immunotherapy, cancer

## Abstract

**Background:**

Impaired DNA damage response (DDR) can affect immune checkpoint inhibitors (ICI) efficacy and lead to heightened immune activation. We assessed the impact of pathogenic or likely pathogenic (P/LP) germline DDR mutations on ICI response and toxicity.

**Materials and methods:**

A retrospective analysis of 131 cancer patients with germline DNA testing and ICI treatment was performed.

**Results:**

Ninety-two patients were DDR-negative (DDR-), and 39 had ≥1 DDR mutation (DDR+). DDR+ patients showed higher objective response rates (ORRs) compared to DDR- in univariate and multivariable analyses, adjusting for age and metastatic disease (62% vs. 23%, unadjusted OR = 5.41; 95% CI, 2.41-12.14; adjusted OR 5.94; 95% CI, 2.35-15.06). Similar results were seen in mismatch repair (MMR), DDR pathways with intact MMR (DDR+MMRi), and homologous recombination (HR) subgroups versus DDR- (adjusted OR MMR = 24.52; 95% CI 2.72-221.38, DDR+MMRi = 4.26; 95% CI, 1.57-11.59, HR = 4.74; 95% CI, 1.49-15.11). DDR+ patients also had higher ORRs with concurrent chemotherapy (82% vs. 39% DDR-, p=0.03) or concurrent tyrosine kinase inhibitors (50% vs. 5% DDR-, p=0.03). No significant differences in immune-related adverse events were observed between DDR+ and DDR- cohorts.

**Conclusion:**

P/LP germline DDR mutations may enhance ICI response without significant additional toxicity.

## Introduction

The number of patients being treated with immune checkpoint inhibitors (ICIs) is growing rapidly (1). This is in part due to the ever-expanding list of approved indications for ICIs across cancer types, and the realization that patients can achieve durable responses, even in the setting of metastatic disease (2). Outcomes have improved for many types of cancer, and patients are living longer as a result (1, 2). However, this paradigm shift in the management of cancer has not been without cost. A significant number of patients still fail to derive any benefit from this transformative class of drugs, and moreover, a whole new type of toxicity directly attributable to ICIs has been recognized, termed immune-related adverse events (irAEs) (3–5). These limitations continue to hamper the best efforts to improve survival with drugs that have for the most part been more tolerable than conventional therapies.

As a result, there has been continued interest in the discovery of biomarkers that can predict which patients are most likely to benefit from ICIs or develop substantial toxicity. Programmed death-ligand 1 (PD-L1) expression, deficient mismatch repair (dMMR), microsatellite instability (MSI), and tumor mutational burden (TMB) have emerged as clinically relevant predictive biomarkers for ICI efficacy across tumor types (6–13). Biomarkers for toxicity have also been studied, but the supporting data are limited (14). As a result, they are not routinely used in clinical practice.

DNA damage response and repair (DDR) gene alterations have also been linked to improved ICI efficacy (15–21). Distinct biologic mechanisms have been proposed explaining the predictive capacity of DDR alterations, including increased neoantigen presentation, increased PD-L1 expression, increased TMB, and increased pro-inflammatory cytokine release (notably interferon-gamma) triggered by activation of the stimulator of interferon genes (STING) and mitochondrial antiviral signaling protein (MAVS) pathways (22–24). These features provide a strong biologic rationale in support of DDR gene alterations as clinical biomarkers. DDR gene alterations are also common across many cancer types, and they can be detected with next-generation sequencing (25). As a result, they are frequently encountered in clinical practice. However, the degree to which individual DDR gene or pathway alterations are predictive of ICI outcomes remains unknown.

The primary purpose of our study was to explore the effects of pathogenic/likely pathogenic (P/LP) germline DDR mutations on ICI efficacy. While less common than somatic alterations, P/LP germline alterations are estimated to occur at a frequency of 4.2-13.8% of all cancers (26). We posited that germline mutations would be more likely to impact the response to ICI treatment than somatic alterations, because germline mutations are present in all tumor cells (in comparison to a fraction of the cells with somatic mutations). Due to the rare nature of germline mutations, we chose to combine all cancer types in our cohort. We hypothesized that like TMB, dMMR, MSI, and PD-L1, DDR mutations would be tissue agnostic in their predictive capacity. To this end, we investigated the effects of deleterious germline DDR mutations in a retrospective single institution study of patients treated with ICIs.

## Materials and methods

### Patients and study design

After institutional review board approval, we retrospectively identified all adult patients (age ≥ 18 years) who had germline DNA sequencing and were also treated with an immune checkpoint inhibitor at the University of California San Diego between September 1st, 2014 and September 1^st^, 2021. 165 patients were identified. Clinical data was then extracted from the electronic medical record. We excluded patients who did not have measurable disease at the start of therapy (n=13) and patients without post-treatment imaging (n=21), producing a cohort of 131 patients. Patients were not required to have metastatic disease and may have been treated with ICIs in the neoadjuvant or palliative setting. All cancer types were allowed. Patients were eligible for inclusion regardless of whether they received ICIs as standard-of-care treatment or while participating in a clinical trial.

The primary objective of this analysis was to examine whether germline P/LP DDR gene mutations were associated with objective response rate [ORR; complete response (CR) + partial response (PR)] by investigator-assessed radiographic response according to RECIST v1.1 (27). A stratified analysis of P/LP DDR subgroups [homologous recombination (HR), mismatch repair (MMR), and DDR altered with intact MMR genes (DDR+MMRi)] was performed after a significant overall association was found. Secondary objectives included a stratified analysis by treatment type, the frequency and type of irAEs, and the effect of P/LP DDR gene mutations on irAEs.

### Sample collection and processing

Blood or saliva was collected and shipped to a Clinical Laboratory Improvement Amendments (CLIA)-certified commercial entity for next-generation DNA sequencing. Gene panels for germline mutations were tested per standard of care at the time of sample receipt, and germline testing may have occurred at any time point within the study period. The number of genes tested varied according to practices of each commercial entity at the time of testing and patient risk factors for inherited syndromes. Incidental DDR germline mutations that were discovered by comparing the genomic signature of solid tumor tissue samples with blood or saliva were included in this analysis (n=10).

PD-L1 immunohistochemistry was performed on formalin fixed paraffin embedded tissue (FFPE). Any commercially-available anti-PD-L1 antibody was allowed. PD-L1 positivity was quantified as a percentage of staining on either tumor or immune cells. The PD-L1 percentages reported are thus a mixture of tumor proportion scores and combined proportion scores. When both tumor proportion scores and combined proportion scores were reported for a subject, the higher value was included for analysis. TMB-high was defined as ≥ 10 mutations/megabase. dMMR/microsatellite instability (MSI)-high was defined by the loss of MLH1, MSH2, MSH6, or PMS2 expression via immunohistochemistry on FFPE tissue (dMMR) or by the detection of unstable microsatellite regions using next-generation sequencing of FFPE tissue or blood (MSI-high).

### DDR genes and determination of P/LP mutation status

A total of 20 genes were identified as being associated with DDR and were grouped into different functional pathways from published resources (**Supplementary Table S1**) (17, 19, 21, 28). We considered all loss-of-function alterations deleterious, including nonsense mutations, splice site, deletions spanning multiple exons, and frameshift alterations (**Supplementary Table S2**). All mutations were manually reviewed in the ClinVar database. DDR+ was defined as any P/LP mutation in one of the 20 DDR genes listed in **Supplementary Table S1** according to the commercial entity performing the sequencing. DDR- patients did not meet this criterion.

### Immune-related adverse events

irAEs were recorded by the treating physician and graded per the American Society of Clinical Oncology guidelines as grades 1–4 (29). There was one death attributable to myasthenia gravis. This was considered a grade 5 irAE.

### Data collection

Study data were collected and managed using REDCap electronic data capture tools hosted at the University of California San Diego (30, 31). REDCap (Research Electronic Data Capture) is a secure, web-based software platform designed to support data capture for research studies, providing 1) an intuitive interface for validated data capture; 2) audit trails for tracking data manipulation and export procedures; 3) automated export procedures for seamless data downloads to common statistical packages; and 4) procedures for data integration and interoperability with external sources.

### Ethics approval and consent to participate

This study was approved by the University of California San Diego institutional review board (ID: #150348CX). Patient consent was not applicable. This study was performed in accordance with the Declaration of Helsinki.

### Statistics

Comparisons of DDR- vs DDR+ groups were analyzed by chi-square or Fisher’s exact tests for categorical data. Continuous variables were compared using independent t-tests for normally distributed samples and the Mann-Whitney U Test for samples without a normal distribution. ORRs were compared using multiple logistic regression. Clinical variables with a p-value < 0.10 with univariate analysis were included in the regression model to address potential confounding (age, metastatic disease, ± TMB; dMMR/MSI-high was not included in the regression models because P/LP gene mutations in MMR genes usually result in dMMR and high MSI).

## Results

### Patient characteristics

**Figure 1:**
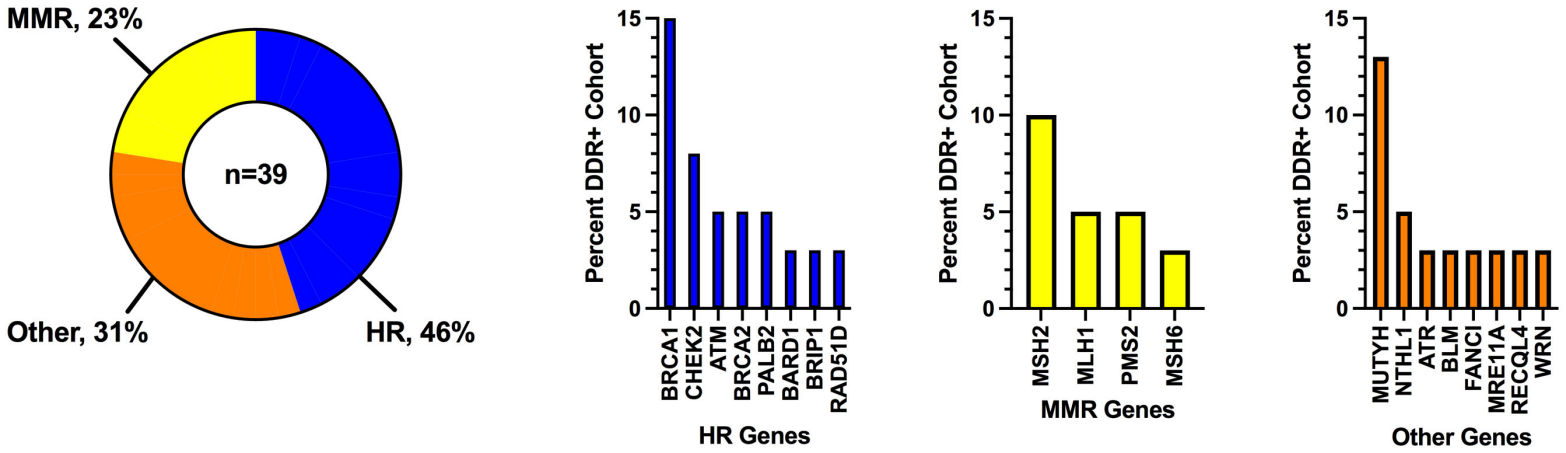
Frequency of DNA damage response mutations grouped by pathway. DDR, DNA damage response; HR, homologous recombination; MMR, mismatch repair.

A total of 131 patients with germline mutation testing and subsequent treatment with ICI at the University of California San Diego between January 1^st^, 2014 and September 1^st^, 2022 were included in the analysis. 117 patients were tested with a commercial germline panel (most commonly 157 gene panel), and 14 patients had pathogenic/likely pathogenic germline mutations identified on paired blood samples that accompanied tumor samples at commercial tumor profiling laboratories. Thirty-nine patients (30%) had a P/LP germline mutation in a DDR pathway, of which 18 (46%) had mutations in the homologous recombination pathway, 9 (23%) had mutations in the mismatch repair pathway, and 12 (31%) had mutations in other DDR pathways (**Figure 1**; see **Supplementary Tables S1**, **S2** for DDR pathway groupings and specific gene mutations). The most common gene mutations were: *BRCA1* (n=6, 15%), *MUTYH* (n=5, 13%), *MSH2* (n=4, 10%), and *CHEK2* (n=3, 8%). Eight mutations were identified in genes that are included on large cancer germline panel tests but for which single gene mutations are not typically associated with an increased risk of cancer; these are genes associated with recessive conditions in which individuals with homozygous or compound heterozygous mutations may have an increased risk of cancer as part of the disease phenotype (*ATR, BLM, FANCI, MRE11A, NTHL1, RECQL4*, and *WRN*).

Patient characteristics are shown in **Table 1**. The majority of patients identified as non-Hispanic (79%) and White (66%). The mean age was 54 years. Eighty-eight percent of patients had metastatic disease. Variables with a statistically significant difference between the DDR- and DDR+ groups included: mean age (52 vs 60 years, p<0.01), TMB (10% vs 26% TMB-High, p=0.03), and dMMR/MSI-High (6% vs 28%, p<0.01). Cancer types were grouped as follows: gastrointestinal (37%), breast (27%), genitourinary (16%), gynecologic (4%), melanoma (4%), neuroendocrine/adrenal (4%), and other (8%) (see **Supplementary Figure S1** for a complete list of cancer types).

**Table 1:** Patient Characteristics [mean (SD) or n (%) reported].

	All patients (n=131)	DDR- (n=92)	DDR+ (n=39)	p-value
Mean age, years	54 (15)	52 (13)	60 (14)	<0.01
Female sex	76 (58)	51 (55)	25 (64)	0.44
Ethnicity				0.34
Hispanic	25 (19)	17 (19)	8 (21)	
Non-Hispanic	103 (79)	74 (80)	29 (74)	
Not documented	3 (2)	1 (1)	2 (5)	
Race				0.55
Asian	8 (6)	6 (7)	2 (5)	
Black	5 (4)	3 (3)	2 (5)	
Other or mixed	30 (23)	20 (22)	10 (26)	
White	87 (66)	63 (69)	24 (61)	
Not documented	1 (1)	0 (0)	1 (3)	
Tobacco smoking status				0.99
Former or current	40 (31)	28 (30)	12 (31)	
Never	88 (67)	62 (68)	26 (67)	
Not documented	3 (2)	2 (2)	1 (2)	
ECOG performance status				0.66
0	46 (35)	30 (33)	16 (41)	
1	67 (51)	47 (51)	20 (51)	
2	12 (9)	10 (11)	2 (5)	
3	4 (3)	3 (3)	1 (3)	
Not documented	2 (2)	2 (2)	0 (0)	
Cancer type				0.66
Breast	35 (27)	24 (26)	11 (28)	
Gastrointestinal	48 (37)	31 (34)	17 (44)	
Genitourinary	21 (16)	17 (19)	4 (10)	
Gynecologic	6 (4)	4 (4)	2 (5)	
Melanoma	6 (4)	4 (4)	2 (5)	
Neuroendocrine/adrenal	5 (4)	3 (3)	2 (5)	
Other^a^	10 (8)	9 (10)	1 (3)	
Metastatic disease	115 (88)	84 (91)	31 (80)	0.08
PD-L1 immunohistochemistry^b^				0.73
< 1%	18 (14)	13 (14)	5 (13)	
1 - 49%	32 (24)	23 (25)	9 (23)	
≥ 50%	14 (11)	8 (9)	6 (15)	
Not documented	67 (51)	48 (52)	19 (49)	
TMB				0.03
High^c^	19 (14)	9 (10)	10 (26)	
Low	73 (56)	57 (62)	16 (41)	
Not documented	39 (30)	26 (28)	13 (33)	
dMMR or MSI-High				<0.01
No	93 (71)	72 (78)	21 (54)	
Yes	16 (12)	5 (6)	11 (28)	
Not documented	22 (17)	15 (16)	7 (18)	
Treatments				
Immune checkpoint inhibitor				0.78
Anti-PD-(L)1	105 (80)	74 (80)	31 (80)	
Anti-CTLA-4	1 (1)	1 (1)	0 (0)	
Anti-PD-1 + anti-CTLA-4	25 (19)	17 (19)	8 (20)	
Line of therapy	2 (1–10)	2 (1–10)	2 (1–7)	0.16
Chemotherapy				
Concurrent	34 (26)	23 (25)	11 (28)	0.83
Prior	80 (61)	58 (63)	22 (56)	0.56
Tyrosine kinase inhibitor				
Concurrent	25 (19)	19 (21)	6 (15)	0.63
Prior	18 (14)	14 (15)	4 (10)	0.58

a Other: 2 cancers of unknown primary, 3 head and neck, 2 lung cancer, 2 mesothelioma, and 1 thyroid cancer.

b PD-L1 IHC was defined as percent tumor cell staining or tumor-associated immune cell staining.

c TMB high was defined as a TMB ≥ 10 mutations/megabase.

DDR, DNA damage response; dMMR, deficient mismatch repair; ECOG, Eastern Cooperative Oncology Group; MSI, microsatellite instability; TMB, tumor mutational burden; (+) indicates the presence of a pathogenic/likely pathogenic mutation.

Most patients (80%) received anti-PD1 or anti-PD-L1 ICIs, with the remainder receiving combined anti-PD-1 plus anti-CTLA-4 (19%) and one patient (1%) receiving single-agent anti-CTLA-4 (**Table 1**). The median line of therapy was 2 (range 1-10). A majority of patients (61%) received prior chemotherapy, and 26% received it concurrent with ICI. Fourteen percent of patients were previously treated with a tyrosine kinase inhibitor (TKI), while 19% received TKI therapy concurrent with ICI. There were no significant treatment differences between DDR- and DDR+ groups.

### Association of P/LP germline mutations with clinical benefit

**Figure 2:**
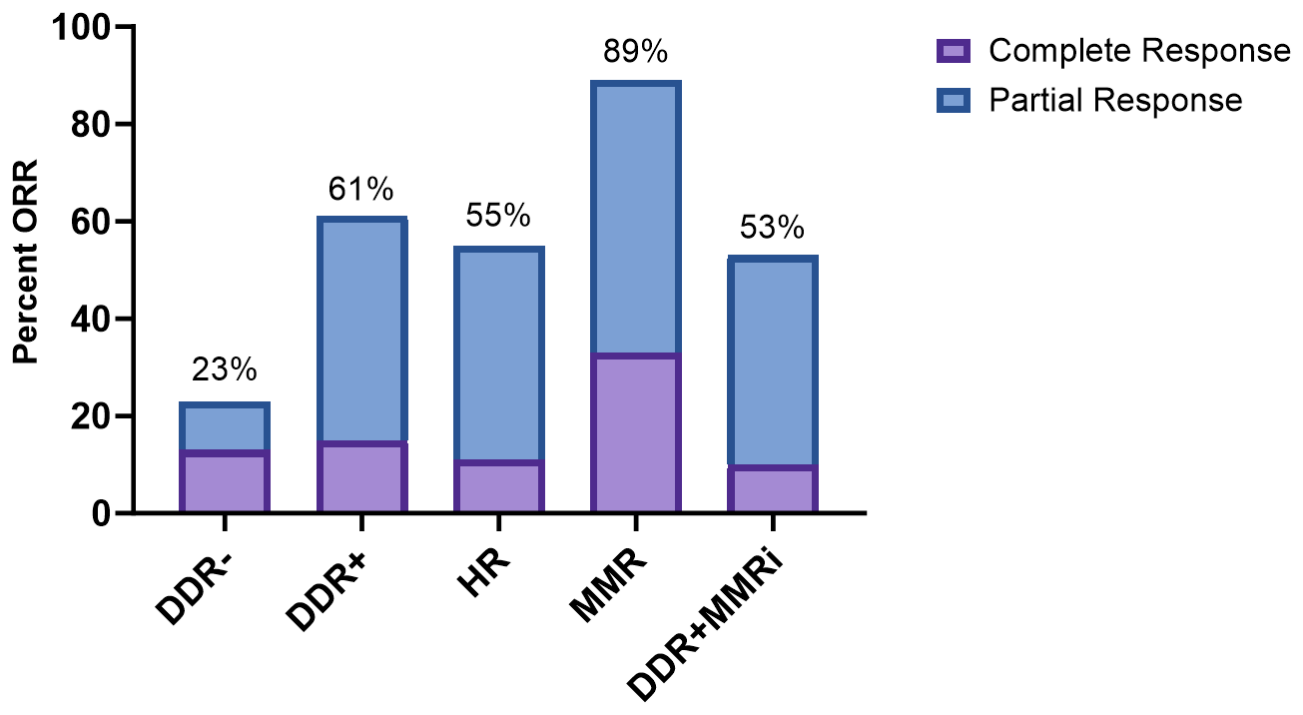
Response to immune checkpoint inhibition. DDR, DNA damage response; HR, homologous recombination; MMR, mismatch repair; MMRi, mismatch repair intact; (+) indicates the presence of a pathogenic/likely pathogenic mutation.

In the entire cohort of 131 patients treated with ICIs, 45 patients (35%) achieved an objective response (**Supplementary Table S3**). The ORR was 61% for DDR+ patients vs 23% for DDR- patients (**Figure 2** and **Table 2**). This was statistically significant (unadjusted odds ratio = 5.41; 95% CI, 2.41 to 12.14). All examined subsets had statistically improved ORRs compared to the DDR- cohort in the unadjusted analysis (HR: odds ratio = 4.23; 95% CI, 1.48-12.07; MMR: odds ratio = 27.05; 95% CI, 3.20-228.77; DDR+MMRi: odds ratio = 3.86; 95% CI, 1.62-9.19).

**Table 2:** Association of germline P/LP DDR+ mutations and objective response.

	ORR, n (%)	Unadjusted		Adjusted ^a^	
		Odds Ratio (95% CI)	p-value	Odds Ratio (95% CI)	p-value
DDR- (n=92)	21 (23)	–	–	–	–
DDR+ (n=39)	24 (62)	5.41 (2.41-12.14)	<0.001	5.94 (2.35-15.06)	<0.001
MMR (n=9)	8 (89)	27.05 (3.20-228.77)	<0.01	24.52 (2.72-221.38)	<0.01
DDR+MMRi (n=30)	16 (53)	3.86 (1.62-9.19)	<0.01	4.26 (1.57-11.59)	<0.01
HR (n=18)	10 (56)	4.23 (1.48-12.07)	<0.01	4.74 (1.49-15.11)	<0.01

a Adjusted for age and metastatic disease.

CI, confidence interval; DDR, DNA damage response; HR, homologous recombination; MMR, mismatch repair; MMRi, mismatch repair intact; ORR, objective response rate; P/LP, pathogenic/likely pathogenic; (+) indicates the presence of a P/LP mutation.

In a multivariable analysis adjusted for age and metastatic disease, DDR+ patients were more likely to achieve an objective response than DDR- patients (adjusted odds ratio = 5.94; 95% CI, 2.35-15.06; **Table 2**). This was also true for all tested subgroups (adjusted odds ratio HR: 4.74; 95% CI, 1.49-15.11; MMR: 24.52; 95% CI, 2.72-221.38; DDR+MMRi: 4.26; 95% CI, 1.57-11.59). We next examined how the addition of TMB would affect the adjusted regression model (**Supplementary Table S4**). The DDR+ cohort and all subgroups continued to have significantly higher odds of achieving an objective response with this model.

Objective response rates by individual P/LP germline DDR mutations are described in **Supplementary Figure S2A**. Eight of the 9 patients with MMR mutations (*MLH1, MSH2, MSH6*, and *PMS2*) achieved an objective response; all 9 patients were also found to have dMMR by immunohistochemistry or MSI-High by next-generation sequencing. Unadjusted odds ratios were calculated for subgroups with at least 3 samples. There was a signal for higher response rates in patients with *BRCA1, CHEK2*, and *MUTYH* genes, but none of these reached statistical significance.

Higher response rates were also noted for all DDR+ cancer types excluding kidney, melanoma and ovarian cancer (**Supplementary Figure S1B**). This was likely due to the small sample size of DDR+ kidney and ovarian cancers (both n=1), and the impressive 100% ORR in melanoma regardless of DDR status. It is also worth noting the difference in ORR for patients with DDR- vs DDR+ breast cancer (25% vs 55%), particularly because none of these patients had MMR mutations [also note that the percent of patients with breast cancer in DDR- vs DDR+ cohorts was nearly the same, 26% vs 28% (**Table 1**), and not statistically different, reducing the likelihood of confounding]. Unadjusted odds ratios were calculated for subgroups with at least 3 samples, but again none of these reached statistical significance.

The effect of germline P/LP DDR mutations on objective response was also assessed in a stratified analysis by treatment type (**Table 3**) and compared to other biomarkers (**Table 4**). Notably, DDR+ patients were more likely to have an objective response compared to DDR- patients regardless of whether they received ICIs with no concurrent therapy, ICIs concurrent with chemotherapy, or ICIs concurrent with TKI. The adjusted odds ratios for these respective groups were as follows: 5.00 (95% CI, 1.53-16.35), 38.85 (95% CI, 2.15-701.74), and 18.95 (0.79-455.65). The adjusted odds ratio for the concurrent TKI group did not reach statistical significance, but there was an impressive difference in ORR for the small number of patients included (DDR- 5% vs DDR+ 50%, adjusted ORR 18.95 (95% CI, 0.79-455.65). PD-L1 positivity (1-50% and >50%), TMB-high, and dMMR/MSI-High each predicted higher odds of achieving an objective response, but only PD-L1 ≥ 50%, high TMB and dMMR/MSI-High were statistically significant in our cohort. The unadjusted odds ratios were: 8.75 (95% CI, 1.76-43.60) for PD-L1 ≥ 50%, 4.87 (95% CI, 1.67-14.19) for high TMB, and 8.63 (95% CI, 2.54-29.31) for dMMR/MSI-High. PD-L1 1-50% did not reach statistical significance (unadjusted OR 2.1; 95% CI, 0.56-7.88).

**Table 3:** Effect of germline P/LP DDR+ mutations on objective response stratified by therapy.

	ORR, n (%)	Unadjusted		Adjusted ^a^	
		Odds Ratio (95% CI)	p-value	Odds Ratio (95% CI)	p-value
No concurrent therapy (n=74)					
DDR- (n=50)	11 (22)	–	–	–	–
DDR+ (n=24)	14 (58)	4.96 (1.73-14.21)	<0.01	5.00 (1.53-16.35)	<0.01
Concurrent chemotherapy (n=34)					
DDR- (n=23)	9 (39)	–		–	
DDR+ (n=11)	9 (82)	7.00 (1.22-40.12)	0.03	38.85 (2.15-701.74)	0.01
Concurrent TKI (n=25)					
DDR- (n=19)	1 (5)	–		–	
DDR+ (n=6)	3 (50)	18.00 (1.38-235.69)	0.03	18.95 (0.79-455.65)	0.07

a Adjusted for age and metastatic disease.

CI, confidence interval; DDR, DNA damage response; ORR, objective response rate; P/LP, pathogenic/likely pathogenic; TKI, tyrosine kinase inhibitor; (+) indicates the presence of a P/LP mutation.

**Table 4:** Association of PD-L1, TMB, and dMMR/MSI-high and objective response.

	ORR, n (%)	Unadjusted Odds Ratio (95% CI)	p-value
PD-L1 < 1% (n=18)	4 (22)	–	–
PD-L1 1-50% (n=32)	12 (38)	2.1 (0.56-7.88)	0.27
PD-L1 > 50% (n=14)	10 (71)	8.75 (1.76-43.60)	<0.01
TMB-low (n=73)	19 (26)	–	–
TMB-high (n=19)	12 (63)	4.87 (1.67-14.19)	<0.01
MSS (n=93)	24 (26)	–	–
dMMR/MSI-high (n=16)	12 (75)	8.63 (2.54-29.31)	<0.001

CI, confidence interval; dMMR, deficient mismatch repair; MSI, microsatellite instability; MSS, microsatellite stable; NS, not significant; ORR, objective response rate; TMB, tumor mutational burden.

### Association of germline pathogenic mutations with irAEs

No significant differences in the rates of irAEs were found between DDR+ and DDR- patients, or between DDR+ patient subgroups, in univariate or multivariable analysis (**Supplementary Figure S3**; **Supplementary Table S5**). DDR- patients had an irAE rate of 51%, while DDR+ patients had an irAE rate of 56%. The frequency, type, and severity of irAEs were also similar between DDR- and DDR+ cohorts regardless of treatment (ICI monotherapy, ICI with concurrent chemotherapy, or ICI with concurrent TKI) (**Supplementary Figures S3A–D**).

## Discussion

In this study, we demonstrate that P/LP germline mutations in DDR genes are associated with improved clinical outcomes with ICIs. This extended to our subgroup analyses, which included mutations in MMR, HR, and DDR altered with intact MMR pathways. Most notably, ORRs were higher in the DDR+ cohort and all examined subgroups. We also showed that the strength of association between P/LP germline DDR mutations and objective response with ICIs was similar to that of PD-L1, TMB and dMMR/MSI biomarkers in this tissue-agnostic cohort. Additionally, we found that the improved odds of an objective response in the DDR+ cohort persisted regardless of whether the patients were treated with ICIs alone or in combination with chemotherapy or TKIs. Furthermore, we did not detect any signal of increased toxicity in the DDR+ cohort or any examined subgroups.

To the best of our knowledge, this is the first study to report outcomes with ICIs in a tissue-agnostic cohort of patients with germline P/LP DDR mutations outside of the MMR or polymerase proofreading pathways (15). Our analysis included genes involved with HR, base-excision repair, and other less common pathways. Our findings identify a group of patients who are more likely to benefit from ICIs, and this has important clinical implications. First, as we move forward in an era of personalized medicine, patients and clinicians increasingly want to know the unique risk/benefit profile for a given treatment at an individual level. This is particularly important for ICIs, since the response rates with monotherapy can be lower than conventional therapies (32), and toxicities can be life-threating, particularly when involving the pulmonary, cardiac, or neurologic systems (3–5). Our results show that germline DDR mutations can predict response to ICIs without any added risk of toxicity. This information can therefore be leveraged by the patient and clinician when attempting to select the optimal treatment approach.

Our results also raise important questions for clinical trial design. For example, should studies be designed specifically for patients with DDR mutations? This could be particularly useful for cancers that have traditionally been resistant to ICIs. Furthermore, the pairing of ICIs with targeted agents against DDR pathways [such as poly-ADP-ribose-polymerase (PARP) inhibitors] could be advantageous, and this is an area of active investigation across tumor types (33). Should germline or somatic DDR mutations be established as biomarkers analogous to PD-L1, TMB, or dMMR/MSI? There is a growing body of research that supports this (15–21), and even more tailored subgroups such as HR deficiency could be considered. Notably, the DDR+, HR, and DDR+MMRi P/LP germline mutations were all better predictors of ICI response than PD-L1 1-50%, and performed similarly to TMB, MSI-high, and PD-L1 ≥ 50% in this cohort of patients. Furthermore, the ability of DDR+ gene mutations to predict ICI response was shown to be independent of TMB in our adjusted analysis. We acknowledge, however, that our results are limited by the retrospective nature of this study and inherent selection bias, so prospective studies are needed to answer these questions definitively.

Additionally, we want to highlight that our study was conducted across a broad range of tumor types. This tissue-agnostic approach matches well with the design of many clinical trials, particularly basket studies for immunotherapy. A more detailed analysis did note higher response rates for patients with DDR+ breast cancer (55% vs 25% DDR-), *BRCA1* mutations (50% vs 23% DDR-), and *MUTYH* mutations (40% vs 23% DDR-), but none of these subgroups reached statistical significance due to the small sample sizes. Larger studies are therefore needed to verify these results.

We also found it of great interest that DDR+ mutations were highly effective predictors of response to ICI when combined with other therapies. In our study, DDR+ patients treated with concurrent chemotherapy or concurrent TKIs were more likely to respond to ICIs than DDR- patients (82% vs 39% and 50% vs 5% respectively). Higher odds ratios were also observed in the DDR+ cohorts compared to the DDR- cohorts when treated with concurrent therapy as opposed to ICIs alone, and all DDR+ patients treated concurrently with chemotherapy or TKI were able to achieve stable disease or an objective response. This may be partially explained by the increased sensitivity of DDR+ cancers to platinum agents (34–37), but there could also be a yet unexplained biologic mechanism that makes DDR+ cancers exquisitely sensitive to ICI combination therapies. This idea is supported by a recent publication that failed to show any improved outcome with TKIs when the DDR+ cases were compared to DDR- cases in the absence of ICI use (38). This is another area of research that requires further attention.

Limitations of our study include the retrospective design, lack of formal and blinded imaging review, selection bias due to non-uniform germline testing across tumor types, variability in gene panels, inclusion of DDR gene mutations that are not predictive of cancer susceptibility when heterozygous (which implies a weaker biologic effect), incomplete biomarker testing, heterogeneity of population, and small sample size of the cohort. The latter significantly limited the depth of data analysis, and although we observed signals of benefit in individual DDR+ tumor types and individual DDR genes, we were unable to show statistically significant differences. We are hopeful that as germline testing becomes more routine in clinical practice and is captured in larger databases, this level of resolution will be possible.

In summary, we found that P/LP germline DDR mutations are associated with higher rates of objective response across tumor types without significantly increased toxicity. Our findings remained consistent when adjusting for clinical variables and in stratified analyses by treatment type. Importantly, we were also able to show that P/LP germline DDR mutations remained predictive of treatment response when MMR mutations were excluded from the analysis (the DDR+MMRi subgroup), and in a more restrictive cohort of HR genes. We conclude that P/LP germline DDR mutations can serve as a valuable biomarker for response to immune checkpoint inhibition.

## Data availability statement

The original contributions presented in the study are included in the article/**Supplementary Material**. Further inquiries can be directed to the corresponding author.

## Ethics statement

The studies involving humans were approved by University of California San Diego Institutional Review Board. The studies were conducted in accordance with the local legislation and institutional requirements. The ethics committee/institutional review board waived the requirement of written informed consent for participation from the participants or the participants’ legal guardians/next of kin because the study posed minimal risk to subjects and it was retrospective in nature.

## Author contributions

MD: Conceptualization, Data curation, Formal analysis, Writing – original draft, Writing – review & editing. SB: Conceptualization, Data curation, Formal analysis, Writing – original draft, Writing – review & editing. LM: Conceptualization, Formal analysis, Writing – original draft, Writing – review & editing. MP: Formal analysis, Writing – original draft, Writing – review & editing. HC: Formal analysis, Writing – original draft, Writing – review & editing. SP: Conceptualization, Formal analysis, Writing – original draft, Writing – review & editing.
