# Supplementary material for: Germline DNA damage response gene mutations as predictive biomarkers of immune checkpoint inhibitor efficacy
Source: Front Immunol. 2024 Jan 29;15:1322187. doi: 10.3389/fimmu.2024.1322187 (PMC10859432; doi:10.3389/fimmu.2024.1322187)
Supplement: Supplementary file 1 [file DataSheet_1.docx]

**Supplementary Tables and Figures**

| Table S1. DDR genes and functional pathways | | | | |  |  |
| --- | --- | --- | --- | --- | --- | --- |
| DDR+ | **HR** | **MMR** | **DDR+MMRi** | **Other** |  |  |
| ATM | ATM | MLH1 | ATM | ATR |  |  |
| ATR | BARD1 | MSH2 | ATR | BLM |  |  |
| BARD1 | BRCA1 | MSH6 | BARD1 | FANCI |  |  |
| BLM | BRCA2 | PMS2 | BLM | MRE11A |  |  |
| BRCA1 | BRIP1 |  | BRCA1 | MUTYH |  |  |
| BRCA2 | CHEK2 |  | BRCA2 | NTHL1 |  |  |
| BRIP1 | PALB2 |  | BRIP1 | RECQL4 |  |  |
| CHEK2 | RAD51D |  | CHEK2 | WRN |  |  |
| FANCI |  |  | FANCI |  |  |  |
| MLH1 |  |  | MRE11A |  |  |  |
| MRE11A |  |  | MUTYH |  |  |  |
| MSH2 |  |  | NTHL1 |  |  |  |
| MSH6 |  |  | PALB2 |  |  |  |
| MUTYH |  |  | RAD51D |  |  |  |
| NTHL1 |  |  | RECQL4 |  |  |  |
| PALB2 |  |  | WRN |  |  |  |
| PMS2 |  |  |  |  |  |  |
| RAD51D |  |  |  |  |  |  |
| RECQL4 |  |  |  |  |  |  |
| WRN |  |  |  |  |  |  |
| DDR, DNA damage response; HR, homologous recombination; MMR, mismatch repair; DDR+MMRi, DDR altered with intact mismatch repair genes | | | | | |  |

**Table S2.** List of the 40 deleterious DDR mutations (table S2 uploaded as separate file)


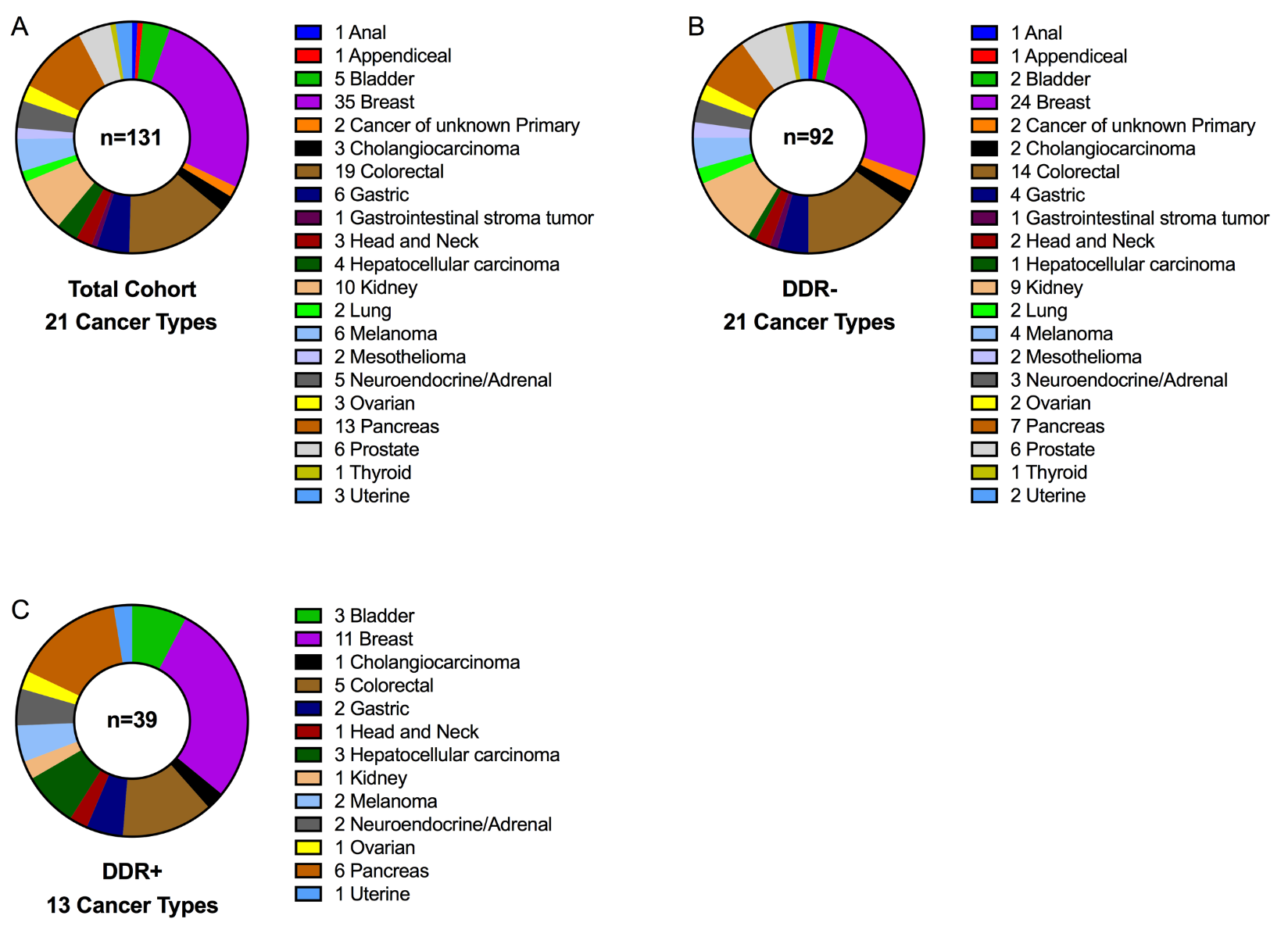


**Figure S1.** Cancer types. Total cohort (A), DDR- cohort (B), and DDR+ cohort (C). DDR, DNA damage response

| **Table S3**. Response to immune checkpoint inhibition, n (%) | | | | | | |
| --- | --- | --- | --- | --- | --- | --- |
|  | CR | PR | SD ≥ 6 months | PD | ORR | DCR |
| All patients (n=131) | 18 (14) | 27 (21) | 16 (12) | 49 (37) | 35% | 47% |
| DDR- (n=92) | 12 (13) | 9 (10) | 13 (14) | 43 (47) | 23% | 37% |
| DDR+ (n=39) | 6 (15) | 18 (46) | 3 (8) | 6 (15) | 61% | 69% |
| MMR (n=9) | 3 (33) | 5 (56) | 1 (11) | 0 (0) | 89% | 100% |
| DDR+MMRi (n=30) | 3 (10) | 13 (43) | 2 (7) | 6 (20) | 53% | 60% |
| HR (n=18) | 2 (11) | 8 (44) | 2 (10) | 3 (17) | 55% | 65% |
| CR, complete response; DCR, disease control rate; DDR, DNA damage response; HR, homologous recombination; MMR, mismatch repair; MMRi, mismatch repair intact; ORR, objective response rate; PD, progressive disease; PR, partial response; SD, stable disease; (+) indicates the presence of a pathogenic/likely pathogenic mutation | | | | | | |

| Table S4. Association of germline P/LP DDR mutations and ORR with adjustments for TMB | | | | | |
| --- | --- | --- | --- | --- | --- |
|  |  | Unadjusted | | Adjusted ^a^ | |
|  | ORR, n (%) | Odds Ratio (95% CI) | p-value | Odds Ratio, (95% CI) | p-value |
| DDR- (n=92) | 21 (23) | - | - | - | - |
| DDR+ (n=39) | 24 (62) | 5.41 (2.41-12.14) | <0.001 | 4.58 (1.51-13.86) | <0.01 |
| MMR (n=9) | 8 (89) | 27.05 (3.20-228.77) | <0.01 | 20.18 (1.94-210.44) | 0.01 |
| DDR+MMRi (n=30) | 16 (53) | 3.86 (1.62-9.19) | <0.01 | 3.96 (1.43-10.93) | <0.01 |
| HR (n=18) | 10 (56) | 4.23 (1.48-12.07) | <0.01 | 3.99 (1.21-13.14) | 0.02 |
| ^a^ Adjusted for age, metastatic disease, and TMB  CI, confidence interval; DDR, DNA damage response; HR, homologous recombination; MMR, mismatch repair; MMRi, mismatch repair intact; ORR, objective response rate; P/LP, pathogenic/likely pathogenic; TMB, tumor mutational burden; (+) indicates the presence of a P/LP mutation | | | | | |

**
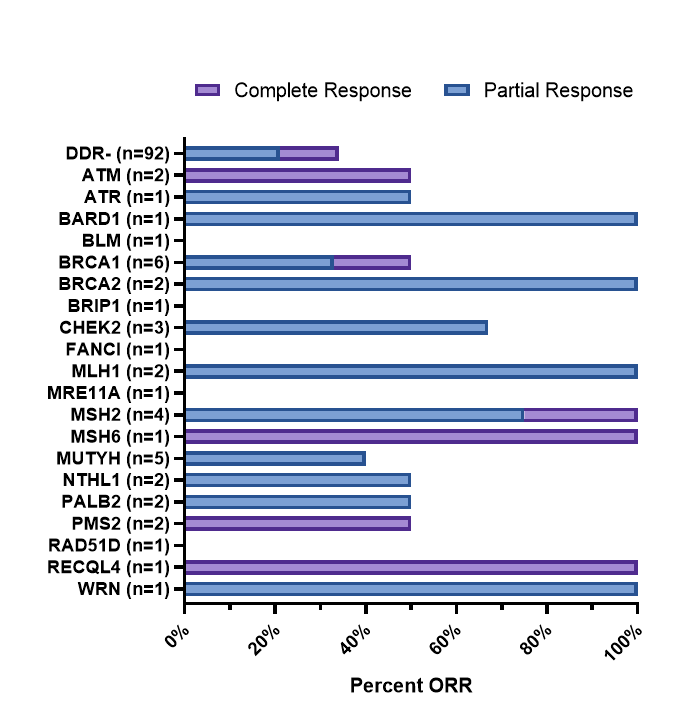
**

B

A


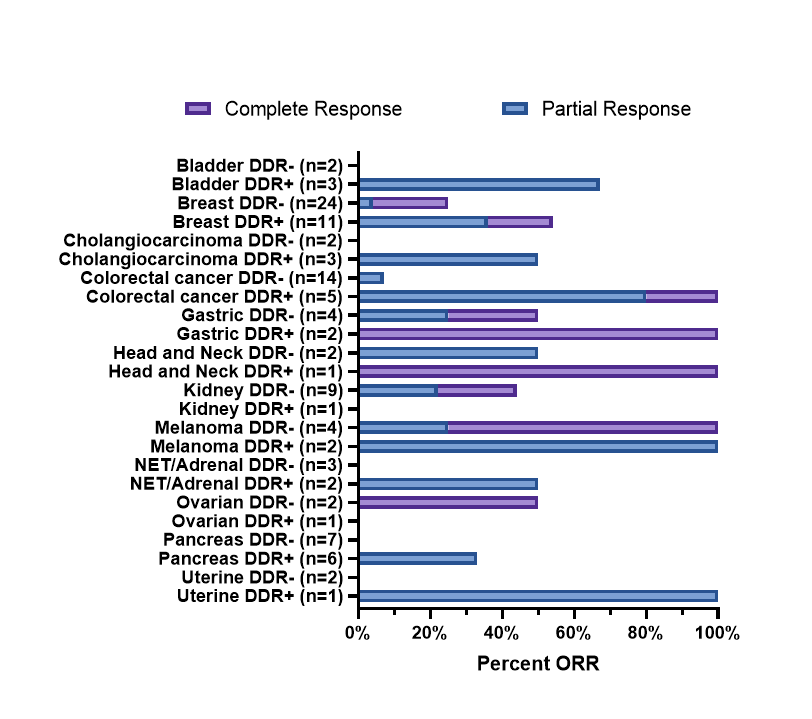


dMMR/MSI-H

**Figure S2.** Objective response rate by gene (A) and cancer type (B). dMMR, deficient mismatch repair; DDR, DNA damage response; MSI, microsatellite instability; (+) indicates the presence of a P/LP mutation.


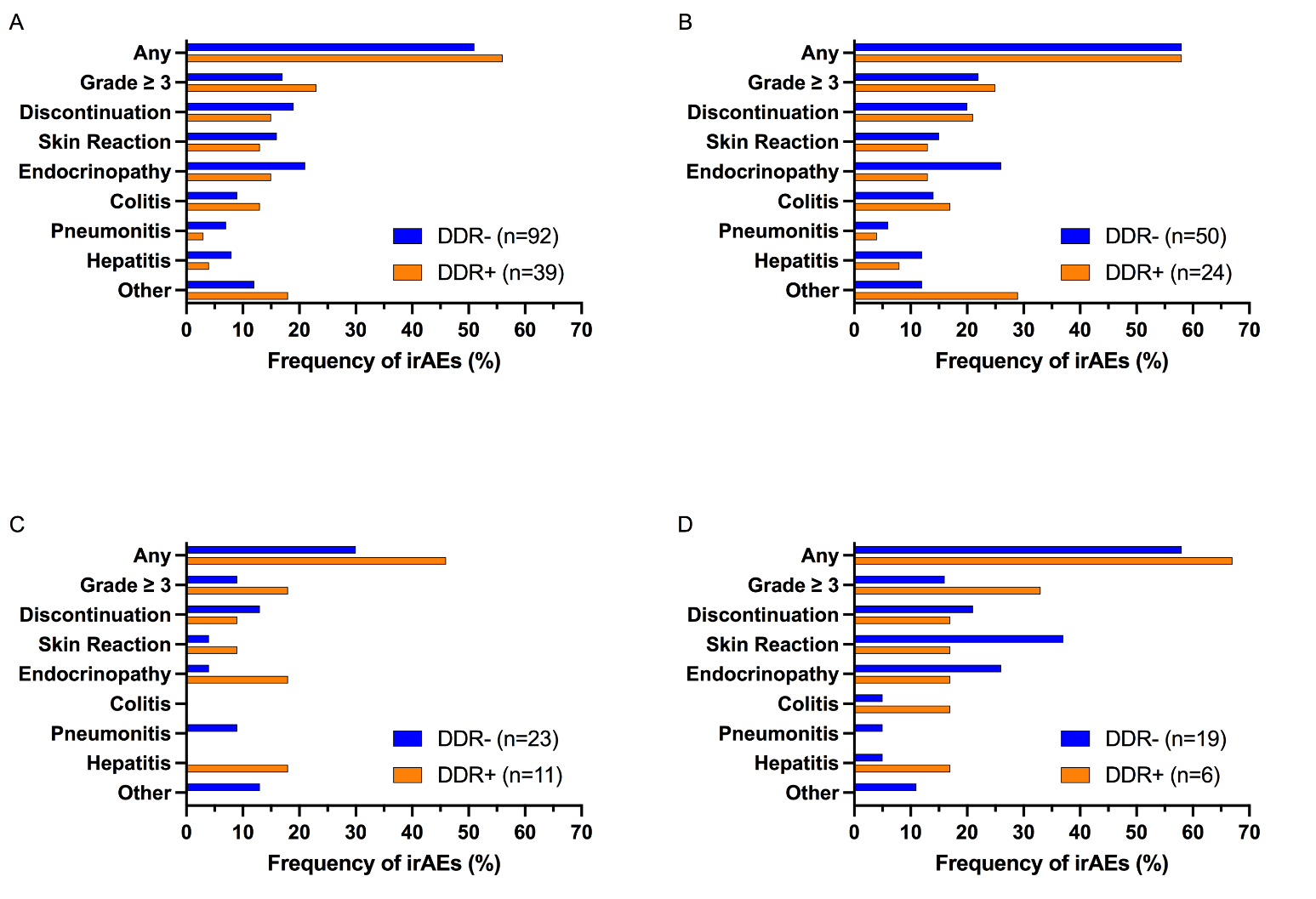


**Figure S3.** Comparison of irAEs between patients with and without P/LP DDR gene mutations in the total cohort (A), no concurrent treatment group (B), concurrent chemotherapy group (C), and concurrent tyrosine kinase inhibitor group (D). DDR, DNA damage response; irAE, immune-related adverse event; P/LP, pathogenic/likely pathogenic; (+) indicates the presence of a P/LP mutation.

| Table S5. Association of germline P/LP DDR mutations and irAEs | | | | |
| --- | --- | --- | --- | --- |
|  | irAE  n (%) | Unadjusted  Odds Ratio (95% CI) | Adjusted^a^  Odds Ratio (95% CI) |  |
| DDR- (n=92) | 47 (51) | - | - |  |
| DDR+ (n=39) | 22 (56) | 1.24 (0.58-2.63) | 1.30 (0.59-2.87) |  |
| MMR (n=9) | 6 (67) | 1.92 (0.45-8.12) | 2.08 (0.47-9.19) |  |
| DDR+MMRi (n=30) | 16 (53) | 1.09 (0.48-2.50) | 1.15 (0.49-2.70) |  |
| HR (n=18) | 8 (44) | 0.77 (0.28-2.12) | 0.80 (0.29-2.23) |  |
| ^a^ Adjusted for age and metastatic disease  CI, confidence interval; DDR, DNA damage response; MMR, mismatch repair; HR, homologous recombination; irAE, immune-related adverse event; MMRi, mismatch repair intact; P/LP, pathogenic/likely pathogenic; (+) indicates the presence of a pathogenic mutation | | | | |
